# DIW-Printed Thermal Management PDMS Composites with 3D Structural Thermal Conductive Network of h-BN Platelets and Al_2_O_3_ Nanoparticles

**DOI:** 10.3390/polym16111491

**Published:** 2024-05-24

**Authors:** Hongyi Zhu, Shunxia Wu, Rui Tang, Yang Li, Gang Chen, Bingxue Huang, Biyou Peng

**Affiliations:** School of Materials Science and Engineering, Xihua University, Chengdu 610039, China; 13568950316@163.com (H.Z.); 18783538270@163.com (S.W.); 18782292773@163.com (R.T.); ly448861934@163.com (Y.L.); gangchen@mail.xhu.edu.cn (G.C.)

**Keywords:** boron nitride, Al_2_O_3_, thermally conductive composites, three-dimensional thermal network

## Abstract

Electronic devices play an increasingly vital role in modern society, and heat accumulation is a major concern during device development, which causes strong market demand for thermal conductivity materials and components. In this paper, a novel thermal conductive material consisting of polydimethylsiloxane (PDMS) and a binary filler system of h-BN platelets and Al_2_O_3_ nanoparticles was successfully fabricated using direct ink writing (DIW) 3D printing technology. The addictive manufacturing process not only endows the DIW-printed composites with various geometries but also promotes the construction of a 3D structural thermal conductive network through the shearing force during the printing process. Moreover, the integrity of the thermal conductive network can be optimized by filling the gaps between the BN platelets with Al_2_O_3_ particles. Resultingly, the configuration of the binary fillers is arranged by the shearing force during the DIW process, fabricating the thermal conductive network of oriented fillers. The DIW-printed BN/Al_2_O_3_/PDMS with 45 wt% thermal conductive binary filler can reach a thermal conductivity of 0.98 W/(m·K), higher than the 0.62 W/(m·K) of the control sample. In this study, a novel strategy for the thermal conductive performance improvement of composites based on DIW technology is successfully verified, paving a new way for thermal management.

## 1. Introduction

The rapid evolution of electronic device technology, particularly within the semiconductor-integrated circuit industry, has become a cornerstone of the modern economy. This progression, characterized by escalating operational frequencies and intensifying workloads, has inadvertently triggered a substantial rise in heat generation within these sophisticated systems [1,2,3]. The accumulation of this heat poses a profound threat to the operational safety, performance reliability, and long-term durability of electronic devices, rendering the development of advanced heat dissipation strategies an imperative aspect of contemporary device engineering [4,5]. Historically, thermal management strategies have relied heavily on effective metal or ceramic-based solutions, which often struggle to reconcile the evolving demands of the electronics sector. Factors such as flexibility, lightweight construction, and broad accessibility have emerged as critical requirements that traditional thermal conductivity devices have found challenging to accommodate. Consequently, there has been a growing interest in thermal conductive composites that can strike a balance between thermal performance and the current trend of electronic devices [6,7].

Polymer materials, celebrated for their combination of lightweight construction, high specific strength, electrical insulation properties, ease of processing, and robust chemical stability, have become ubiquitous across multiple industries, including electronics, biotechnology, energy, and manufacturing [8]. Despite these advantages, their suboptimal thermal conductivity has confined their application scope to non-performance-critical areas, such as packaging, within electronic devices [9,10]. To address this limitation, researchers have turned to incorporating high-thermal-conductivity fillers into polymer matrices, a strategy aimed at boosting thermal conductivity without sacrificing other beneficial polymer properties [11,12]. In the field of thermal material research and development, commonly used fillers such as aluminum oxide (Al_2_O_3_), zinc oxide (ZnO), and boron nitride (BN) have been proven to process the ability to significantly optimize the thermal conductivity of composite materials [13]. The optimization effect of thermal conductive fillers on the thermal conductivity of materials mainly depends on the construction of a three-dimensional thermal conductive network [14,15]. To meet the thermal conductive requirement, a high filler loading would usually be needed to fabricate an adequate thermal conductive network. However, the quest for optimal thermal conductivity often necessitates high filler loadings, which can lead to a reduction in the polymer matrix’s mechanical integrity, dielectric properties, and rheological behavior [16,17,18]. This underscores the importance of developing strategies that promote efficient bonding between thermal conductive fillers and the polymer matrix while simultaneously reducing the overall filler content, a delicate balance crucial for the successful integration of these materials into electronic devices.

Direct ink writing (DIW) is a transformative additive manufacturing technology that has revolutionized the fabrication of complex 3D structures from a myriad of materials, including polymers, metals, ceramics, and composites [19]. The essence of DIW lies in its inks, characterized by a pronounced shear-thinning behavior that ensures smooth passage through narrow nozzles and preservation of the intended shape after deposition [20]. This technology has gained significant traction due to its compatibility with a wide range of materials, environmentally friendly printing process, and its proven track record in constructing functional devices in the biomedical, water treatment, and flexible electronics fields [21,22,23]. In addition to the excellent structure-forming capacity and material compatibility, it is noteworthy that the shear stress field generated during DIW printing has a significant regulatory effect on the arrangement of fillers in the ink [24]. A notable example of DIW’s potential in thermal management is illustrated by Yang et al., who engineered a flexible thermal management device using glass fibers within a rubber matrix. By precisely tuning the orientation of glass fibers under the influence of shearing forces exerted by the DIW printer’s extrusion needle, they achieved a highly interconnected thermal conductive network with minimal filler usage [25]. This approach highlights the potential for optimizing thermal conductivity through filler manipulation and strategic material placement.

To further push the boundaries of thermal management solutions, our research delves into a novel strategy combining DIW 3D printing with the synergistic effects of binary fillers in the context of PDMS-based composites. PDMS, renowned for its flexibility and electrical insulation capabilities, has faced limitations in widespread adoption due to its inherently low thermal conductivity [26]. In this paper, based on the PDMS matrix, a strategy for the construction of a thermal conduction network of binary fillers (BN nanoflakes and Al_2_O_3_ particles) has been demonstrated, taking advantage of DIW 3D printing technology. Following this strategy, BN/Al_2_O_3_/PDMS polymer composites were DIW-printed to prepare a thermal management device with improved thermal conductivity. Within the direct ink writing (DIW) methodology, the microstructure of boron nitride (BN) nanosheets was manipulated under shearing forces, culminating in the assembly of a directionally aligned thermal conductor filler configuration. Additionally, the interstice between these BN nanosheets can be filled with aluminum oxide (Al_2_O_3_) nanoparticles, which serve to refine the structure and enhance the continuity of the thermal conduction network. To validate this design principle, the establishment of an efficacious thermal conduction network was achieved. Notably, PDMS composites reinforced with 45 wt% BN/Al_2_O_3_ exhibited a thermal conductivity of 0.98 W/(m·K), accompanied by a robust tensile strength of 1.35 MPa and exceptional electrical insulation characteristics. These findings underscore the potential of DIW-fabricated BN/Al_2_O_3_/PDMS composites in revolutionizing thermal management strategies for electronic devices. This study effectively demonstrates a sophisticated approach for manufacturing high-efficiency thermal management systems, thereby outlining a promising avenue for advancing the thermal performance of contemporary electronics.

## 2. Materials and Methods

### 2.1. Materials

BN platelets with an average size of 1–2 µm and Al_2_O_3_ nanoparticles were purchased from Shanghai Aladdin Biochemical Technology Co., Ltd. (Shanghai, China), and PDMS precursors (Sylgard 184) and the corresponding curing agent (Octamethylcyclotetrasiloxane) were supplied by Dow Corning Corporation, Midland, MI, USA. All materials were used as received.

### 2.2. Synthesis of BN/Al_2_O_3_/PDMS

**Figure 1:**
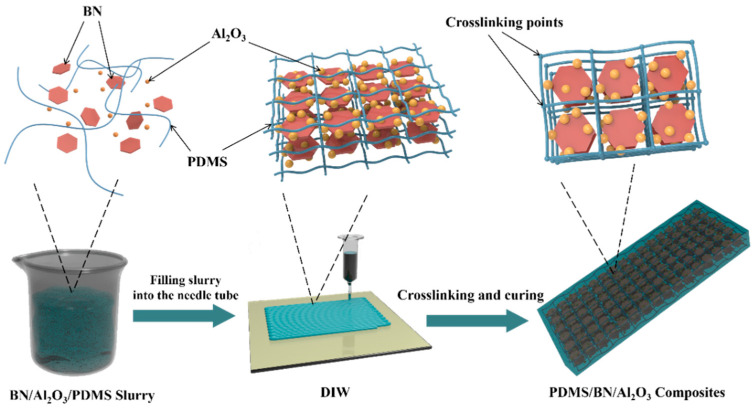
Schematic of the DIW ink preparation process for BN/Al_2_O_3_/PDMS composites.

The preparation process of the BN/Al_2_O_3_/PDMS composites is shown in Figure 1. The PDMS precursor and curing agent were added to a beaker according to the mass ratio of 10:1 and stirred under mechanical stirring at 800 r/min for 2 min. The solution was left at room temperature for 20 min, and then a certain amount of micronized BN and Al_2_O_3_ were added into the configured solution and mechanically stirred at 500 r/min for 5 min. The prepared slurries were printed on a DIW 3D printer (HKing-D2, Hanjing Space Digital Technology Co., Ltd., Chengdu, China) at a speed of 600 mm/min, and the printed parts were named 40 BN/Al_2_O_3_, 40 BN, and 45 BN/Al_2_O_3_, respectively. The detailed slurry formulations are shown in Table 1. The DIW printed parts were then cured in an oven at 80 °C for 2 h.

### 2.3. Characterization

The rheological properties of the slurries for DIW printing were measured using a rotational rheometer (MCR 302, Anton Paar, Graz, Austria). Viscosity tests were performed in the range of 0.01–100 s^−1^ shear rate. Frequency scanning tests were performed in oscillatory mode with a frequency range of 0.1–100 Hz. The yield stress was measured in shear stress scanning mode with a shear stress range of 1–1000 Pa. The orientation property of the binary fillers within the PDMS composites was measured using an X-ray diffractometer (DX-2700 BH, Haoyuan Instrument Co. Ltd., Dandong, China) of Cu Kα radiation (λ = 0.154 nm) at ambient temperature with a scanning range of 10 to 60 °C. The cross-sectional morphology and microstructure of the DIW-printed parts were observed using a field emission scanning electron microscope (Hitachi SU8010, Hitachi, Tokyo, Japan). In addition, an infrared thermal imager (FOTRIC 220 RD, FOTRIC, Shanghai, China) was used to evaluate the thermal response and thermal management capabilities of the samples. A Keithley 2601B was used to measure the volume resistivity of the printed samples. Dielectric constant and dielectric loss were determined using an Agilent LCR analyzer.

The thermal conductivity measurements were executed using a thermal constants analyzer (TPS 2200, Hot Disk, Hot Disk AB, Uppsala, Sweden) operating in a transient anisotropic module, conforming to the specifications outlined in standard ISO/CD 22007-2 [27,28]. The calculation of thermal conductivity (*k*) was performed based on the following equation:

*k* = *α* × *ρ* × *Cp* (1)

Here, *α* signifies the thermal diffusivity measured in square meters per second (m^2^/s), *ρ* represents the density of the composite material given in grams per cubic centimeter (g/cm^3^), and *Cp* denotes the specific heat capacity, determined by means of a differential scanning calorimeter (TA-DSC 25, TA Instruments, New Castle, DE, USA), expressed in joules per gram Kelvin (J/g·K). The density (*ρ*) was calculated utilizing the principle of Archimedes. The cylindrical specimens utilized for these tests featured a diameter of 12.6 mm and a thickness of 3 mm, ensuring consistency in sample geometry for accurate and reliable thermal property assessments.

## 3. Results and Discussion

### 3.1. The Rheological Behavior during DIW Printing

**Figure 2:**
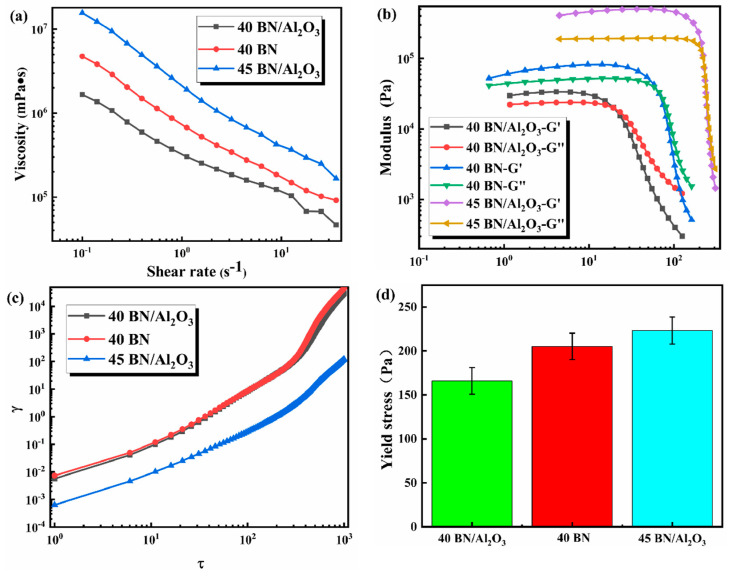
Rheological properties of 40 BN/Al_2_O_3_, 40 BN, and 45 BN/Al_2_O_3_ slurries: (**a**) plots of slurry viscosity versus shear rate; (**b**) plots of modulus and shear stress of slurries; (**c**) plots of stress-strain relationship of slurries; (**d**) plots of yield stress of slurries.

In the typical DIW printing process, appropriate rheological properties are the key to ensuring high DIW printing performance in areas such as printing resolution and structural stability [29]. Therefore, the rheological properties of the BN/Al_2_O_3_/PDMS slurries with different filler contents were systematically characterized, and the results are shown in Figure 2. The BN flakes and Al_2_O_3_ were used as the thermal conductive fillers, which were added to the PDMS precursor to form a white slurry for DIW printing. For a successful DIW printing process, the viscosity of the DIW ink is required to decrease with an increasing shear rate, which allows the DIW ink to flow smoothly through the nozzle under air blast. As shown in Figure 2a, the viscosity of the three prepared slurries all decreased with the increase in shear rate, which is in line with the shear thinning effect of the polymer solution. For the 40 BN/Al_2_O_3_ and 40 BN samples with the same solid content, it could be seen that the viscosity of the 40 BN/Al_2_O_3_ samples was much lower than that of the 40 BN samples at the same shear rate, which should be attributed to that the Al_2_O_3_ would reduce the interaction of the PDMS molecules. The 45 BN/Al_2_O_3_ samples with a higher solid constant demonstrated a higher viscosity than that of 40 BN/Al_2_O_3,_ which could be the result of the stronger interaction between PDMS molecules and the binary fillers. Hence, the viscosity of the prepared slurries would be able to ensure the smooth operation of the DIW printing.

Figure 2b shows the change in storage modulus (G′) and loss modulus (G″) of the three kinds of slurries (40 BN/Al_2_O_3_, 40 BN, and 45 BN/Al_2_O_3_) with the shear stress at the range of 1 × 10^−2^ to 7 × 10^2^ Pa. It is widely accepted that the material elasticity and viscosity can be represented by storage modulus (G′) and loss modulus (G″), respectively. The solid-like behavior of the slurries dominated by elasticity would appear while the value of G′ is higher than that of G″. The slurries in the solid-like state could maintain their structure endowed by the DIW technology, presenting adequate structural stability. Moreover, if the value of G′ is lower than that of G″, the slurries would display liquid-like behavior, allowing the slurries to flow like liquid during the extrusion process. Therefore, the measurement of G′ and G″ is a necessary process during the development of DIW inks. As for the prepared slurries in this paper (40 BN/Al_2_O_3_, 40 BN, and 45 BN/Al_2_O_3_), in the linear viscoelastic region (LVER), the values of G′ were higher than that of G″. Beyond the yield point where the values of G′ and G″ were equal, their strain values suddenly decreased. The LVER of the 40 BN/Al_2_O_3_, 40 BN, and 45 BN/Al_2_O_3_ slurries increased sequentially because of the increasing strength of the network structure enhanced by the binary fillers.

Figure 2c shows a histogram of the yield stress, whose yield stress value C is tested as the ratio of stress to strain at the point of mutation:

(2) $$C=\frac{\tau }{\gamma }$$

where  τ is the shear stress and  γ is the shear strain, and from the figure. The values of the yield stress of the 40 BN/Al_2_O_3_, 40 BN, and 45 BN/Al_2_O_3_ slurries were measured as 168, 206, and 220 Pa, respectively, which presented the same pattern of change as that of viscosity measurement results (Figure 2d). In general, the rheological performance of the prepared slurries was suitable for DIW 3D printing to achieve a stable and smooth printing process.

### 3.2. Analysis of the Printing Quality of DIW Parts

**Figure 3:**
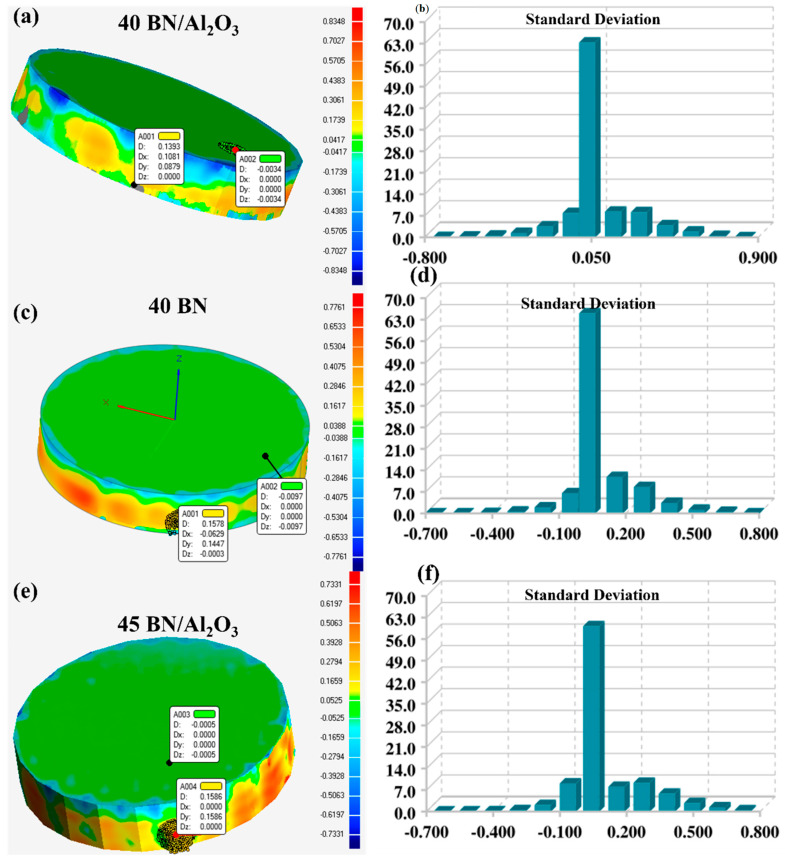
Printing quality analysis of DIW structures: (**a**,**c**,**e**) the error analysis; (**b**,**d**,**f**) the standard deviations of size difference.

For DIW-printed composites, the printing quality is an important impactor of their application potential. Excellent print quality ensures tight connections and uniform distribution between material layers, thereby maximizing thermal conductivity and reducing thermal resistance [30]. Therefore, the printing quality was evaluated by reverse engineering modeling technology, shown in Figure 3. In detail, the DIW print was scanned by a laser 3D scanner to build a digital model with precise size data. The DIW printing quality of the prepared slurries in this paper can be quantified through the difference between the size of the model built by reverse engineering and the original printing model. As for the three DIW-printed BN/Al_2_O_3_, 40 BN, and 45 BN/Al_2_O_3_ structures, the maximum size differences were 0.84 mm, 0.77 mm, and 0.73 mm, respectively, and the largest percentage of the size difference occurred at 0.04, 0.05 and 0.05 mm, respectively. The results show that the prepared slurries of PDMA precursor and thermal conductive fillers in this paper demonstrate satisfactory printing quality. Furthermore, the 45 BN/Al_2_O_3_ slurry demonstrated the smallest size difference among the three samples, proving its outstanding printing quality. This measurement result from the reverse engineering could be explained by the rheological measurement results. The storage modulus of the 45 BN/Al_2_O_3_ slurry exceeded 5 × 10^5^ Pa, which endowed the slurry with the ability to maintain the structure conducted by DIW printing technology.

### 3.3. Microscopic Morphology of Composite Parts

**Figure 4:**
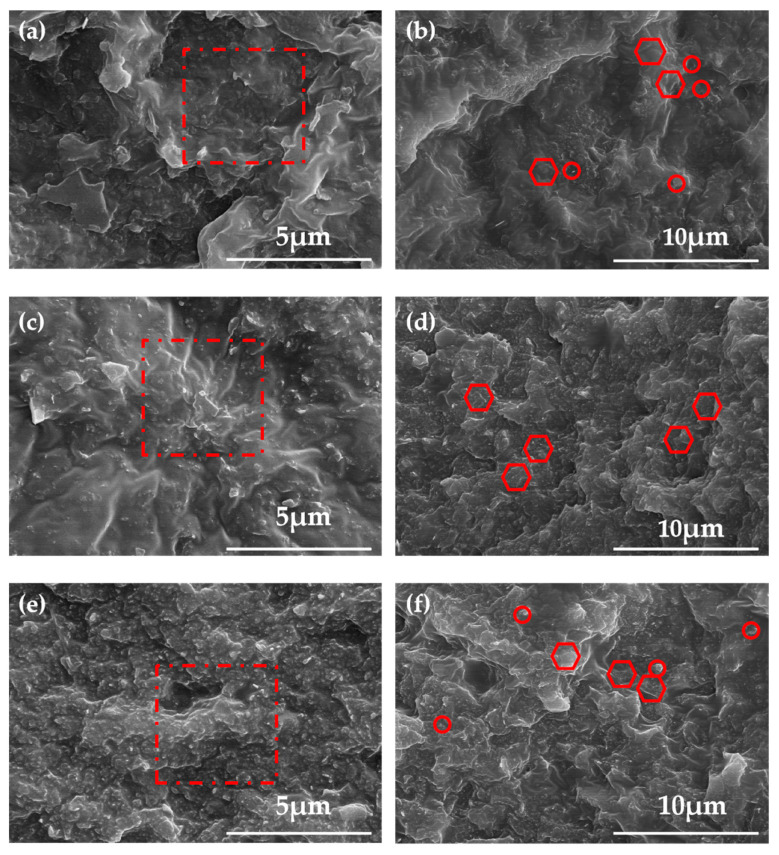
The SEM images of (**a**,**b**) 40 BN/Al_2_O_3_; (**c**,**d**) 40 BN; (**e**,**f**) 45 BN/Al_2_O_3_.

A large number of studies have shown that the overlapping of thermal conductive fillers has a significant impact on the thermal conductivity of the material [31]. The crosssection of the DIW-printed BN/Al_2_O_3_/PDMS composites was observed by SEM to explore the arrangement of fillers in the PDMS matrix (Figure 3). Due to the mechanical characteristics of the PDMS matrix, the DIW-printed PDMS-based composites all presented a rough cross-section, and there were no visible air voids or agglomeration of the filler, proving the compatibility of the PDMS matrix with the binary fillers. During the DIW printing process, the slurry inks experienced an increasing shear force, and the fillers in the ink tended to orient along the direction of the shear force, which lessened the flow resistance during the DIW printing process. The configuration of the fillers could be maintained after the solidification of the slurry ink. As depicted in Figure 4, the SEM images showed few traces of filler. This morphology of the cross-section of 40 BN/Al_2_O_3_ and 40 BN could be because most fillers were wrapped into the PDMS matrix. In contrast, there were abundant morphological features of the filler in the SEM image of the 45 BN/Al_2_O_3_ cross-section, and an obvious orientation of the thermal conductive filler along the in-plane direction could be observed. Therefore, the micromorphology of BN in PDMS can be controlled by the DIW preparation technique, which in turn regulates the properties of the composites.

**Figure 5:**
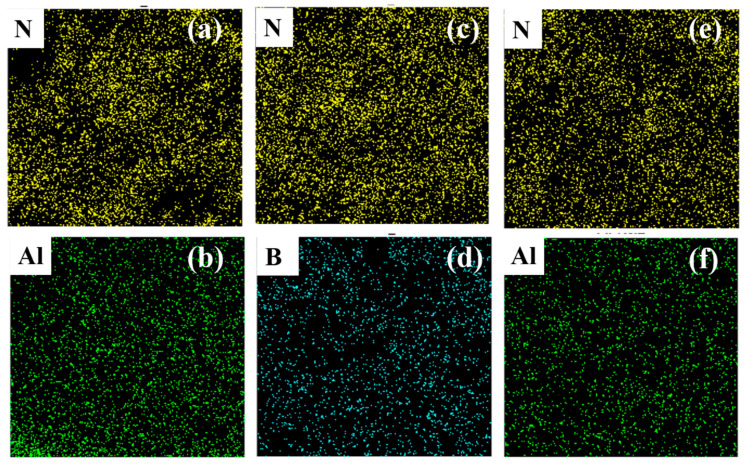
EDS images of different printed parts: (**a**,**b**) 40 BN/Al_2_O_3_; (**c**,**d**) 40 BN; (**e**,**f**) 45 BN/Al_2_O_3_.

The EDS images in Figure 5 demonstrate the Al, B, and N elements distribution in the DIW-printed 40 BN/Al_2_O_3_, 40 BN, and 45 BN/Al_2_O_3_ structures. The distribution of N and Al elements in the EDS images indicates that the BN flakes and Al_2_O_3_ nanoparticles are distributed uniformly in the PDMS matrix, which again verifies the compatibility of the PDMS matrix with the thermal conductive fillers. The uniform distribution of the binary fillers lays a stable foundation for further control measures of filler configuration.

### 3.4. BN Orientation Analysis of Composites

**Figure 6:**
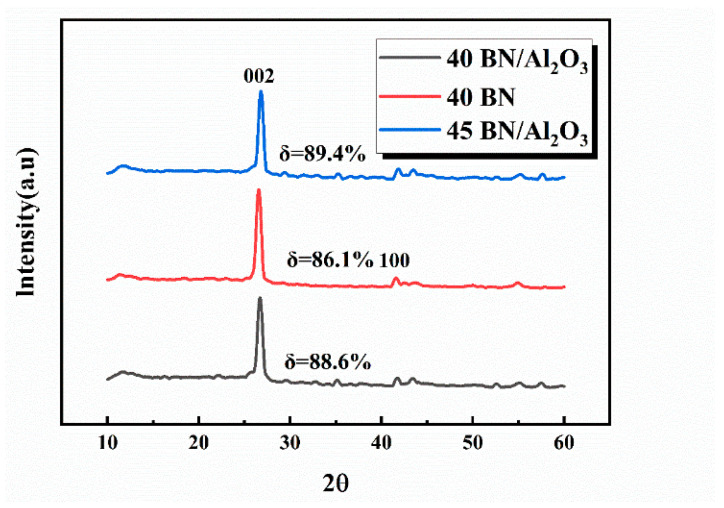
XRD patterns of DIW-printed 40 BN/Al_2_O_3_, 40 BN, and 45 BN/Al_2_O_3_ composites.

To further explore the filler orientation in the PDMS matrix, XRD measurement was employed to quantify the orientation degree. Figure 6 depicts the XRD measurement results of 40 BN/Al_2_O_3_, 40 BN, and 45 BN/Al_2_O_3_ composite materials. As depicted in the XRD pattern, there is a distinct sharp peak at 27°, indicating the typical crystal structure of BN nanoflakes [32]. The degree of orientation δ was taken and calculated using Equation (2) according to the previous relevant work [33]:

(3) $$\delta =\frac{I_{\left(002\right)}}{I_{\left(100\right)}+I_{\left(002\right)}}\times 100\%$$

where *I*(100) and *I*(002) are the intensity of the (100) and (200) peaks on the XRD spectra, respectively. The peaks at around 27.0° and 42.0° corresponded to the (002) and (100) lattice plane of BN nanoflakes. The degree of filler orientation of the DIW-printed 40 BN/Al_2_O_3_, 40 BN, and 45 BN/Al_2_O_3_ composites was all beyond 85%, which can prove the regulating effect of shear force during the DIW printing process. In the comparison of samples 40 BN/Al_2_O_3_ and 40 BN with the same filler content, it can be observed that the 40 BN/Al_2_O_3_ had a higher degree of BN orientation. The 45 BN/Al_2_O_3_ sample was obtained by adding 5 wt% of Al_2_O_3_ to the 40 BN/Al_2_O_3_ sample. It can be seen that the degree of the filler orientation increased with the increase in the solid content of the slurry, which was in line with the SEM measurement results.

### 3.5. Thermal Conductivity of Composite Materials

**Figure 7:**
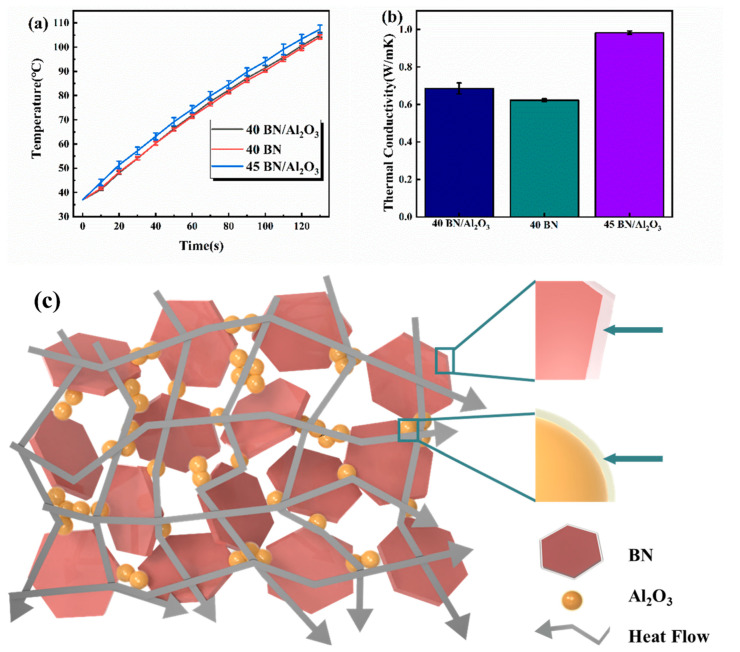
The thermal management of the DIW-printed PDMS-based composites: (**a**) The top surface temperature of the cylindric 40 BN/Al_2_O_3_, 40 BN, and 45 BN/Al_2_O_3_ composite where the bottom surface of these samples was deposited on a heater with a temperature of 200 °C; (**b**) Thermal conductivity of composite materials; (**c**) The heat transfer mechanism model of 45 BN/Al_2_O_3_ composite.

In order to vividly demonstrate the thermal management ability of the PDMS-based composites with the binary fillers, the top surface temperature of the columniform device (diameter: 12.6 mm and height: 3 mm) made of 40 BN/Al_2_O_3_, 40 BN, and 45 BN/Al_2_O_3_ composites in the same environment was recorded, respectively, where the bottom surface of the cylindrical device was deposited on a heater with a temperature of 200 °C. The results are depicted in Figure 7a. After being heated for 130 min, the temperature of 45 BN/Al_2_O_3_ was recorded as 107 °C, which was higher than those of 40 BN/Al_2_O_3_ and 40 BN. It could be observed that the best thermal conductivity performance was obtained from the 45 BN/Al_2_O_3_, proving its superior heat transfer capabilities. In the composites with the same solid content, the temperature change of the 40 BN/Al_2_O_3_ was greater than that of 40 BN, which indicates an improved thermal conductivity achieved by the synergistic effect of the binary fillers. The thermal conductivity of the 40 BN/Al_2_O_3_, 40 BN, and 45 BN/Al_2_O_3_ composites were measured at 0.68, 0.62, and 0.98 W/(m·K) (Figure 7b), respectively, which was in keeping with the thermal management behavior of these three samples in Figure 7a. As reported in the previous work [34], the thermal conductivity of the pure PDMS material was measured at 0.19 W/(m·K). Therefore, it could be concluded that the BN nanoflakes showed an obvious positive influence on the thermal conductive performance of PDMS material. Meanwhile, at the same filler loading, the thermal conductivity of 40 BN/Al_2_O_3_ was improved by 10% compared to that of sample 40 BN. The difference in the thermal conductive behaviors of these samples verifies the effect of binary thermal conductive fillers in improving the thermal conductivity. To better present the thermal conductivity mechanism of the BN/Al_2_O_3_/PDMS composites with binary fillers, a heat transfer schematic was constructed, as shown in Figure 7c. Due to the introduction of spherical Al_2_O_3_ filler, the space between the BN nanoflakes was filled by Al_2_O_3_ spheres, thus improving the integrity of the thermal conductive network. Therefore, a more complete thermal conductivity network was formed in the BN/Al_2_O_3_/PDMS composites, which was more favorable to improving the thermal conductivity of the fabricated parts.

### 3.6. Electrical Insulation Properties of Composites

**Figure 8:**
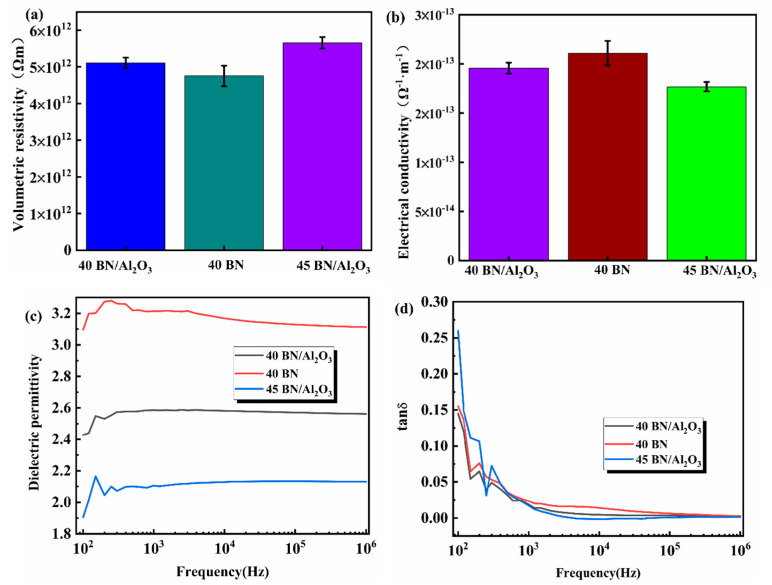
Electric insulation performance of 40 BN/Al_2_O_3_, 40 BN, and 45 BN/Al_2_O_3_; (**a**) volume resistivity; (**b**) conductivity; (**c**) dielectric constant; (**d**) dielectric loss.

According to the common consensus, the dielectric insulation performance of composite materials in electronic devices is crucial. For example, composite materials with low dielectric constant and dielectric loss can withstand faster signal transmission rates and reduce signal transmission attenuation. In order to investigate the electrical insulating properties of the composites under mixed fillers, the volume resistivity, conductivity, dielectric constant, and dielectric loss of the 40 BN/Al_2_O_3_, 40 BN, and 45 BN/Al_2_O_3_ composites were tested systematically, and the results are shown in Figure 8. As reported in recent years, the volume resistivity of electrical insulating material usually exceeded the value of 1 × 10^8^ Ω·m. The volume resistivity of 40 BN/Al_2_O_3_, 40 BN, and 45 BN/Al_2_O_3_ were measured at 5.9 × 10^12^, 4.8 × 10^12^, and 5.7 × 10^12^ Ω·m, respectively, which verified an electrical insulation material with application potential in thermally conductive and electrically insulated devices (Figure 8a,b). As for the prepared composites with the same filler content, the electronic insulating performance of 40 BN/Al_2_O_3_ was higher than that of 40 BN, which could be attributed to the excellent electrical insulation properties of Al_2_O_3_; particles. Furthermore, the dielectric constant and dielectric loss of 40 BN/Al_2_O_3_, 40 BN, and 45 BN/Al_2_O_3_ depicted in Figure 8c,d prove the excellent electrical insulating property as well. At a testing frequency from 10^3^ to 10^6^ Hz, the dielectric loss values of the three samples remain largely stable, indicating the potential application of such materials in high-frequency transmission scenarios. Meanwhile, the dielectric loss of all three materials significantly decreased as the test frequency increased (Figure 8d). The value of the dielectric constant and dielectric loss of 45 BN/Al_2_O_3_ at the frequency range of 10^3^ Hz to 10^6^ Hz both remained at low levels under high-frequency test conditions, which suggests that this material exhibits minimal energy dissipation when subjected to an alternating high-frequency electric field. With these favorable thermal conductive and electrical insulating properties, the BN/Al_2_O_3_/PDMS composites could be promising thermal management devices in the electric area.

### 3.7. Mechanical Properties of Composite Materials

**Figure 9:**
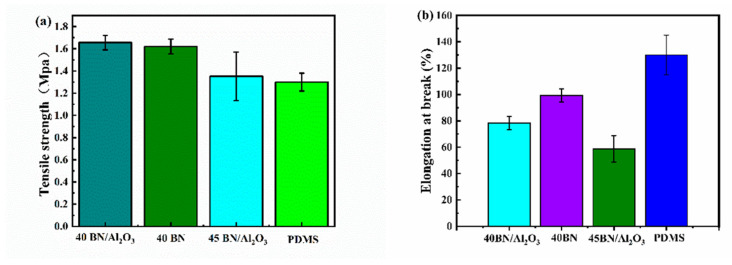
Mechanical properties of the PDMS-based composites (40 BN/Al_2_O_3_, 40 BN, 45 BN/Al_2_O_3_, and pure PDMS matrix): (**a**) tensile strength; (**b**) tensile elongation at break.

A satisfying mechanical performance of the composites was the precondition for the application in electronic devices [35]. In order to study the mechanical flexibility of the composites, the tensile properties of the composites in this paper were tested at a tensile rate of 50 mm/min. The tensile strengths of 40 BN/Al_2_O_3_, 40 BN, and 45 BN/Al_2_O_3_ composites were 1.65, 1.64, and 1.35 MPa, respectively (Figure 9a). Given the tensile strength of the PDMS matrix (1.31 MPa), the mechanical performance of BN/Al_2_O_3_/PDMS composites was remarkably improved due to the strengthening of the binary fillers. The mechanical strength demonstrated an obvious relationship with the thermal conductive filler loading. The tensile breaking strength of samples 40 BN/Al_2_O_3_ and 40 BN was close, while the tensile strength of 45 BN/Al_2_O decreased by 18% compared to 40 BN/Al_2_O_3_. The tensile strength superiority of 40 BN/Al_2_O_3_ over 45 BN/Al_2_O_3_ could be attributed to the stress concentration induced by an excess of filler. Figure 9b shows the tensile elongation of the composites in this paper. The tensile elongation at break of 40 BN/Al_2_O_3_, 40 BN, 45 BN/Al_2_O_3_, and pure PDMS was 78.36%, 99.34%, 58.76%, and 130%, respectively. Obviously, the introduction of thermally conductive fillers would lead to a decrease in the tensile elongation at break of PDMS-based composite materials, which could be the result of the fillers hindering the movement of macromolecular chains. In conclusion, the results show that the addition of thermally conductive fillers not only meets the heat management needs of the material but also provides an optimizing effect on their mechanical performance. It can be seen from the mechanical measurement results that the overloading of the thermal conductive filler to the PDMS matrix could negatively impact the tensile toughness of the composites. Hence, the PDMS-based composites with the highest filler loading of 45 wt% presented the lowest tensile elongation at break among the four tested samples.

## 4. Conclusions

In this paper, a novel strategy for the construction of an efficient thermal conductive network in the matrix of PDMS composite material by DIW printing technology has been successfully proved. The slurries of the PDMS precursor and the thermal conductive agent (BN and Al_2_O_3_) presented proper rheological performance for the DIW printing process, and the DIW-printed slurries could be cured into the BN/Al_2_O_3_/PDMS composite material under heating. Based on this strategy, a BN/Al_2_O_3_/PDMS composite material was fabricated where the binary fillers of BN and Al_2_O_3_ cooperated with the PDMS matrix to achieve satisfying thermal management. With the aid of Al_2_O_3_ nanoparticles, the integrity of the thermal conductive network could be improved where the thermal conduction pathway was mainly constructed from BN flakes, with Al_2_O_3_ spheres filling the gaps between the BN layers like bridges. Moreover, XRD results demonstrated that the arrangement of the binary fillers could be controlled by the shear force during the DIW printing process, achieving the highest orientation degree of 89.4% for the BN and Al_2_O_3_ filler. Based on the merits, the DIW-printed composites of BN/Al_2_O_3_/PDMS reached a satisfying thermal conductivity of 0.98 W/(m·K) with 37 wt% BN flakes and 8 wt% Al_2_O_3_ nanoparticles while their mechanical performance was improved simultaneously, presenting a satisfying tensile strength of 1.35 MPa. The resistance value of the composite material was more than 5.7 × 10^12^ Ω·m, representing an outstanding electrical insulating property. With all of their advantages, the DIW-printed BN/Al_2_O_3_/PDMS composites could pave a new way for the thermal management of electronic devices.

## Figures and Tables

**Table 1:** The slurry formulations for BN/Al_2_O_3_/PDMS composites.

	Al_2_O_3_/wt%	BN/wt%	PDMS Precursor/wt%	Curing Agent/wt%
40 BN/Al_2_O_3_	8.00	32.0	54.0	6.00
40 BN	0.00	40.0	54.0	6.00
45 BN/Al_2_O_3_	8.00	37.0	50.0	5.00

## Data Availability

Data are contained within the article.
